# Relation of Wnt Signaling Pathway Inhibitors (Sclerostin and Dickkopf-1) to Left Ventricular Mass Index in Maintenance Hemodialysis Patients

**DOI:** 10.1155/2021/2439868

**Published:** 2021-09-23

**Authors:** Ahmed Bahie, Mohamed M. Abdalbary, Dalia Younis El-Sayed, Rasha Elzehery, Ghada El-Said, Ghada El-Kannishy, Ahmed M. Abd El Wahab

**Affiliations:** ^1^Internal Medicine Department, Mansoura Nephrology and Dialysis Unit (MNDU), Mansoura Faculty of Medicine, Mansoura, Egypt; ^2^Department of Clinical Pathology, Mansoura University, Mansoura, Egypt

## Abstract

**Background:**

Left ventricular hypertrophy (LVH) is common in hemodialysis (HD) patients. It predicts poor prognosis. Several inhibitors regulate Wnt canonical pathways like Dickkopf-related protein-1 (Dkk-1) and sclerostin.

**Objectives:**

To investigate the relationship between serum sclerostin, Dkk-1, left ventricular mass (LVM), and LVM index (LVMI) in HD patients.

**Methods:**

This is a cross-sectional study including 65 HD patients in our HD unit. Patients were divided into two groups according to LVMI (group 1 with LVMI < 125 gm/m^2^ (*N* = 29) and group 2 with LVMI > 125 gm/m^2^ (*N* = 36)). Echocardiographic evaluation of the LVM, aortic, and mitral valves calcification (AVC and MVC) was done. Serum levels of sclerostin and Dkk-1 and patients' clinical and biochemical data were recorded.

**Results:**

Group 2 showed significantly higher age, blood pressure, AVC, and MVC and significantly lower hemoglobin, sclerostin, and Dkk-1 levels. LVM and LVMI had a significant linear negative correlation to both serum sclerostin and Dkk-1 (*r* = −0.329 and −0.257, *P*=0.01 and 0.046 for LVM; *r* = −0.427 and −0.324, *P*=0.001 and 0.012 for LVMI, resp.). Serum Dkk-1 was an independent negative indicator for LVM and LVMI in multiple regression analyses (*P*=0.003 and 0.041 with 95% CI = −0.963 to −0.204 and −0.478 to −0.010, resp.).

**Conclusion:**

Serum sclerostin and Dkk-1 were significantly lower in HD patients with increased LVMI > 125 gm/m^2^, and both had a significant linear negative correlation with LVM and LVMI. Dkk-1 was a significant negative independent indicator for LVM and LVMI in HD patients.

## 1. Introduction

Left ventricular hypertrophy (LVH) is common structural remodeling in patients with end-stage renal disease, and its presence predicts a poor prognosis [1]. Echocardiographic diagnosis of LVH is based on cutoff values developed from formula formed from population-based studies which indexed the left ventricular mass (LVM) to the body surface area (BSA) [2].

Wnt/*β*-catenin signaling pathway is an essential positive regulated signaling network for cardiovascular diseases [3]. *β*-Catenin increases the expression of target genes associated with cell adhesion and involves the regulation of angiogenesis and atherosclerosis [4].

In addition, *β*-catenin plays a crucial role in heart failure caused by afterload-induced cardiac hypertrophy [5]. It interacts with the transforming growth factor-*β* (TGF-*β*) signaling pathway to exacerbate cardiac fibrosis and aggravate chronic heart failure [6].

Several inhibitors regulate the Wnt canonical pathway, among them Dickkopf-related protein-1 (Dkk-1) and sclerostin (Scl) [7]. Sclerostin is a 190-residue glycoprotein, which is expected to contain a cysteine-knot motif and belongs to the DAN/Cerberus family of proteins [8].

The secreted glycoprotein Dkk-1, a member of the Dickkopf family, is known to antagonize Wnt/*β*-signaling by interaction with the LRP5/6 (low-density lipoprotein receptor-related protein 5/6). The Wnt/*β*-catenin signaling pathway is an important mediator in cardiovascular disease and influences inflammation and vascular calcification [9].

Serum sclerostin levels significantly vary with age and are higher in male patients with stage 3b and 4 CKD than in females with the same stages [10]. Previous studies showed that the expression of Wnt/*β*-catenin signaling inhibitors such as sclerostin and Dkk-1 may attenuate further vascular calcification [11, 12].

We aimed to define the relationship between serum sclerostin, Dkk-1, left ventricular mass (LVM), and LVM index (LVM/BSA) in maintenance hemodialysis (HD) patients.

## 2. Subjects and Methods

### 2.1. Study Design

This is a cross-sectional study involving patients on regular HD in MNDU during the period Jan 2016–Jan 2017. 65 patients were recruited. The patients were grouped according to the presence or absence of left ventricular hypertrophy (LVH) calculated by LVM/BSA (LVMI) into two groups, group 1 with LVMI < 125 gm/m^2^ and group 2 with LVMI > 125 gm/m^2^, and the cut point that indicated LV hypertrophy was 125 gm/m^2^ [13]. All patients were undergoing HD three times/week, 4 h/session using bicarbonate-based dialysate with calcium concentrations of 1.5 mmol/L. Exclusion criteria were age <18 or >75 years, HD duration less than 6 months, patients who had a rheumatic valvular disease or underwent prosthetic valve replacement, and cardiomyopathic patients with EF < 40%. All patients were subjected to history taking with stress on age, smoking state, HD duration, cause of ESRD, drug history and current treatment, associated comorbidities, diabetes mellitus, hypertension, chronic liver disease, CVD, surgical history especially previous renal transplantation, and parathyroidectomy. Estimated glomerular filtration rates (eGFRs) were calculated using the Modification of Diet in Renal Disease (MDRD) equation [14]. Physical examination including weight, height, waist, midarm circumference, blood pressure, edema of lower limb, chest, heart, and abdomin, and AV fistula examination was done.

### 2.2. Laboratory

Fasting blood samples were obtained prior to midweek dialysis sessions for measurement of biochemical data and serum sclerostin and serum Dackkopf-1 (Dkk-1) levels. We measured serum sclerostin using the kit that uses a double-antibody sandwich enzyme-linked immunosorbent assay (ELISA) (SunRed ELISA Kit) (PELOBIOTECH GmbH-Am Klopferspitz 19-82152 Planegg, Germany, catalog number 201-12-5418 (96 tests). The sensitivity was 0.175 ng/ml, with a range of assay being 0.2 ng/ml–60 ng/ml. The serum Dkk-1 was measured using double-antibody sandwich enzyme-linked immunosorbent assay kits (SunRed ELISA Kit supplied by PELOBIOTECH GmbH, Planegg, Germany, catalog number 201-12-0631). The sensitivity of kits was 0.412 ng/mL, with a range of assay being 0.5–150 ng/ml.

Biochemical data including complete blood count, serum albumin, corrected serum calcium, phosphorus, iPTH, serum iron, TIBC, serum ferritin, total cholesterol, total triglycerides, high-density lipoprotein (HDL), and serum LDL-c concentration were obtained. Predialysis urea and postdialysis urea were recorded; Kt/v was calculated using the Daugirdas formula [15].

### 2.3. Radiology Assessment

#### 2.3.1. 2D Echocardiography

An expert echocardiographer, who was unaware of the patients' data, performed echocardiographic measurements according to the recommendations of the American Society of Echocardiography [16]. Patients were examined prior to the HD session lying in a left lateral position in a semidark room; a two-dimensional assessment of the aortic valve and mitral valve was done using Medison SonoAce X6 device. Scoring of mitral calcification was done according to the Wilkins calcification [17] and grading of the aortic valve was done according to a previous study by Tenenbaum et al. [18].

LVM was determined by Devereux's formula (LV mass (ASE) = 0.8 (1.04 ([LVIDD + PWTD + IVSTD] 3-[LVIDD] 3)) + 0.6 g. (LVIDD = left ventricular internal diameter in diastole, PWTD = posterior wall thickness in diastole, and IVSTD = interventricular septum thickness in diastole)) [19] and then divided by body surface area (BSA), in order to obtain LVM index [20] expressed in g/m [2]. LVH was defined as increased LVMI [13].

### 2.4. Calculation of LV Myocardial Performance Index (MPI)

MPI was calculated using (ICT + IRT)/ET formula. The mean normal value of the Tei index is 0.39 ± 0.05 for the LV [20]. Mitral inflow was recorded by conventional pulsed Doppler and tissue Doppler to reveal the left ventricular diastolic function by measuring E (early diastolic) velocity, A (late diastolic) flow velocity, also E_1_ (early diastolic) annular velocity, and A_1_ (late diastolic) annular velocity and calculate E/A ratio and E_1_/A_1_ratio, respectively.

### 2.5. Statistical Analysis

The normality of data was first tested with the Shapiro–Wilk test. Qualitative data were presented by frequency tables (frequency and percentages). Quantitative variables were presented by central indices (mean ± standard deviation) for normally distributed variables and median (minimum-maximum) for nonnormally distributed variables. Pearson's correlation was used to correlate continuous normally distributed data while Spearman's correlation was used to correlate ordinal and nonnormally distributed data. We compared nonnormally distributed and ordinal variables between qualitative groups using the Mann–Whitney U test and Kruskal–Wallis H test. All statistical analyses were performed using SPSS version 24 (IBM Corp., Armonk, NY, USA). A *P* value ≤ 0.05 was considered statistically significant.

## 3. Results

Table 1 shows the demographic, clinical, and laboratory characteristics of the whole study (mean age (years) (46.14 ± 16.201), gender (male 55.6% and female 44.4%), dry weight (kg) (73.16 ± 15.89), midarm circumference (cm) (29.52 ± 4.25), waist circumference(cm) (98.21 ± 16.43), BMI (26.53 ± 5.31), BSA (1.82 ± 0.23)). Regarding smoking, current smokers were 6 (18.8%), ex-smokers were 6 (18.8%), and nonsmokers were 20 (62.4%). The HD duration was 35 months (6.00–168). Hypertensive patients were 85.2%, diabetic patients were 13.1%, and coronary artery disease was present in 9.8%. Kt/v was 1.13 ± 0.28, serum albumin (g/dl) was 3.72 ± 0.44, serum calcium (mg/dl) was 8.3 ± 0.92, serum phosphorus (mg/dl) was 4.93 ± 1.58, iPTH (pg/ml) was 49 (16–221), alkaline phosphatase was 145 (11–2152), and HB (gm/dl) was 9.38 ± 1.65.

Table 2 shows a comparative analysis of some demographic and echocardiographic data among the studied two subgroups, which shows a significant increase in mean age in the second group (with LVM/BSA > 125 gm/m^2^) compared with the first subgroup (with LNM/BSA < 125 gm/m^2^) (37.61 ± 14.47 versus 51.74 ± 14.93; *P* value =0.001), a significant increase in systolic blood pressure and diastolic blood pressure in the second subgroup compared with the first subgroup (*P* < 0.05), and a highly significant increase in aortic valve calcification (AVC) and mitral valve calcification (MVC) in the second subgroup compared with the first subgroup (*P* value <0.001; <0.01). There are no significant changes in BMI, BSA, HD duration, DM, HTN, midarm circumference, waist circumference, dry weight, E/A, mitral E wave deceleration, E1/A1, IVRT, IVCT, ET, and MPI between the studied subgroups.

Table 3 shows a comparative analysis of some laboratory data among the studied two subgroups, which shows a highly significant decrease in the serum hemoglobin concentration (gm/dl) in the second subgroup compared with the first subgroup (10.096 ± 1.43 versus 8.92 ± 1.633; *P* value =0 .006) and significant a decrease in the serum sclerostin (ng/ml) and Dkk-1 (pg/ml) in the second subgroup compared with the first subgroup (35.25 (3.40–59.3) versus 7.70 (2.10–60.4) and 63 (12.00–132) versus 33 (13.00–112); *P* value < 0.05 for both), respectively. There are no significant differences in iron, TSAT%, TIBC, albumin, alkaline phosphatase, cholesterol, TGs, HDL, LDL, Kt/V, Ca, PO4, and iPTH between the studied subgroups.

Table 4 shows the correlation between LVM and LVM/BAS and some demographic and laboratory parameters among the studied subgroups, which shows a significant negative correlation between s. sclerostin, serum Dkk-1, and serum HDL with LVM (Rho = −0.329, −0.427 and −0.268; *P* value =0.010, 0.001, and −0.036, resp.), a significant positive correlation of age, BSA, hypertension, aortic valve calcification, mitral valve calcification, and dry weight with LVM (with *P* value = 0.001, 0.027, 0.019, 0.036, 0.001, 0.02, and 0.028, resp.), and a nonsignificant correlation of the other parameters with LVM (*P* > 0.05). Also, there is a significant negative correlation between s. sclerostin, serum Dkk-1, BSA, hemoglobin concentration, serum albumin, midarm circumference, and dry weight with LVMI with LVM (Rho = −0.257, −0.324, −0.305, −0.299, −0.294, −0.423, and −0.286, resp.; *P* value < 0.05 and 0.01), a significant positive correlation of age, aortic valve calcification, and mitral valve calcification with LVM (with *P* value = 0.007, < 0.001, and 0.004), respectively, and a nonsignificant correlation of the other parameters with LVM (*P* > 0.05).

Table 5 shows a linear regression analysis of age, HTN, HDL, AVC, MVC, s. sclerostin, and Dkk-1 as independent predictors for LVM, which shows that hypertension is a positive predictor for LVM (with *P* value <0.01 and confidence interval (CI) = 10.70 to 70.50) and shows also that HDL and s. Dkk-1 are independent negative predictors for LVM (with *P* value < 0.05 and < 0.01) and CI = −2.33 to −0.117 and −0.963 to −0.204, resp.).

Table 6 shows a linear regression analysis of age, HTN, HDL, AVC, MVC, s. sclerostin, and Dkk-1 as independent predictors for LVMI, which shows that hypertension is a positive predictor for LVMI (with *P* value =0.050 and CI = −0.025 to 36.80), while serum Dkk-1 was an independent negative predictor for LVMI (with *P* value < 0.05 and CI = −0.478 to −0.010).

Table 7 shows that iPTH showed a nonstatistically significant weak positive correlation with both sclerostin and Dkk-1 (*P* > 0.05). It showed a significant positive correlation to phosphorus (*r* = 0.41, *P*=0.001). Both AVC and MVC showed a significant positive correlation with age (*r* = 0.55, *P* < 0.0001 and *r* = 0.38, *P*=0.003, resp.). MVC was significantly negatively correlated to total cholesterol and LDL (*r* = −0.27, *P*=0.04 and *r* = −0.28, *p* = 0.03, resp.). Both AVC and MVC showed a significant negative correlation with sclerostin (*r* = −0.47, *P* ≤ 0.0001 and *r* = −0.27, *P*=0.04, resp.) but they were not significantly correlated to Dkk-1, hemoglobin, iron, TSAT, TIBC, ferritin, albumin, calcium, BP, and dialysis duration (*P* > 0.05).

Table 8 shows that both Dkk-1 and sclerostin did not show a significant correlation with different types of hyperparathyroidism treatment, Kt/v, calcium, phosphorus, ALP, TSAT, TIBC, iron, and ferritin. Dkk-1 showed a significant negative correlation with erythropoietin (EPO) treatment (*r* = −0.372, *P*=0.003) while sclerostin showed a nonsignificant negative correlation to EPO treatment.

**Figure 1:**
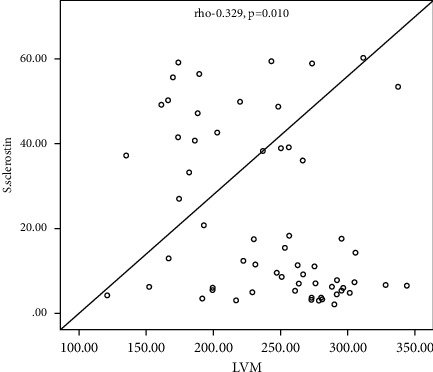
Linear negative correlation between the left ventricular mass and serum sclerostin level.

**Figure 2:**
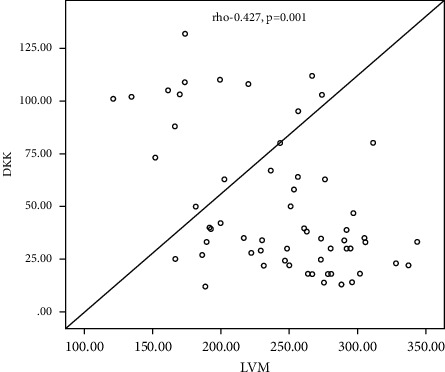
Linear negative correlation between the left ventricular mass and serum Dickkopf-related protein-1.

**Figure 3:**
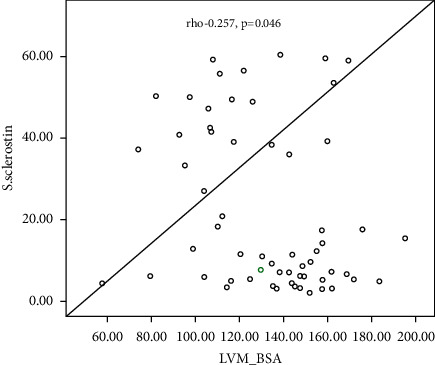
Linear negative correlation between the left ventricular mass index and serum sclerostin level.

**Figure 4:**
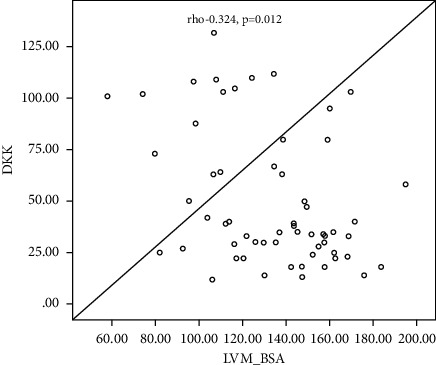
Linear negative correlation between the left ventricular mass index and serum Dickkopf-related protein-1.

**Figure 5:**
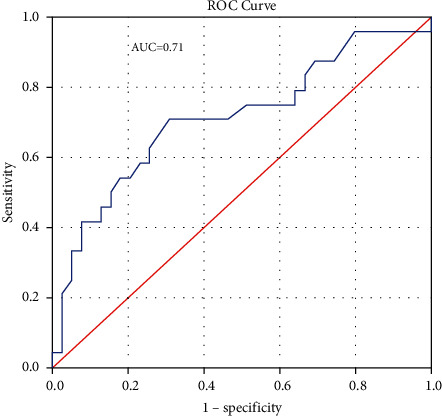
ROC analysis between LVMI and Dkk-1.

Figure 1 shows a linear negative correlation between left ventricular mass and serum sclerostin level.  Figure 2 shows a linear negative correlation between left ventricular mass and serum Dickkopf-related protein-1.  Figure 3 shows a linear negative correlation between left ventricular mass index and serum sclerostin level.  Figure 4 shows a linear negative correlation between left ventricular mass index and serum Dickkopf-related protein-1. In Figure 5, ROC analysis between LVMI and Dkk-1 showed an AUC = 0.71 and *P*=0.005. The cutoff value for Dkk-1 was 38.5, at which sensitivity was 70% and specificity was 70%.

## 4. Discussion

The accumulated data allow us to consider the disturbances in the FGF-23-Klotho-sclerostin ratio as one of the early markers of CKD advancement, disorders of mineral metabolism developing, and cardiovascular prognosis [21]. Alteration in the ratio of FGF-23, serum Klotho, and serum sclerostin can be regarded as an independent early marker of cardiovascular morbidity and overall prognosis of patients with CKD [22].

55.6% of our patients were men with a mean age of 46.1 ± 16.2 years. The majority of them were hypertensive, while diabetes and CAD were present in 13% and 10% of them, respectively. HB concentration was 9.38 ± 1.65 mg/dl. Serum albumin, calcium, and phosphorus were within normal ranges in most patients.

With regard to the comparative analysis of the studied groups, we found a significant increase in age, AVC, and MVC in the second subgroup (with LVM/BSA > 125 gm). A significant increase in systolic and diastolic blood pressures was noted in subgroup 2 despite the nonsignificant difference in the percentage of hypertensive patients in the studied groups which may be attributed to better control of blood pressure and adherence to treatment in group 1 and it also indicates that the second group was subjected to a greater afterload burden. There was a nonsignificant decrease in (E/A ratio) by conventional Doppler study in the subgroup 2 versus subgroup 1 (*P*=0.069; Table 2) denoting more diastolic dysfunction and more increase in the preload in subgroup 2 patients. When tissue Doppler was used, the E1/A1 ratio decreased in group 1 denoting that the tissue Doppler study revealed more patients with diastolic dysfunction in subgroup 1 patients, yet statistically nonsignificant. None of the studied patients in our study had AF. There was a significant increase in hemoglobin concentration in subgroup 1 versus subgroup 2, denoting more malnutrition in subgroup 2.

Also, serum sclerostin and Dickkopf-1 (Dkk-1) levels were significantly lower in subgroup 2 (with LVMI > 125 gm/m^2^) than subgroup 1 (with LVMI < 125 gm/m^2^) which may denote a myocardial protective role for these proteins. On the contrary, Yongqiang Ji et al. reported that high levels of sclerostin in CKD patients (stage 3–5ND) were associated with more valvular calcification [23].

One possible explanation is the start of hemodialysis as it has been reported that, in patients on hemodialysis, the high sclerostin level was negatively correlated with the risk of cardiovascular death and Ji et al. did not include such patients. Chen et al. followed up 84 hemodialysis patients for (12–60 months) and concluded that patients with low baseline serum sclerostin undergoing MHD showed better survival and less CIMT [24]. Zou et al. found no correlation between sclerostin and CVEs in MHD patients [25]. These contradictory results may be attributed to different ethnic groups, different follow-up period, different assessment and measurement of sclerostin, and different primary endpoints. It is known that PTH suppresses the expression of sclerostin and/or Dkk-1 decreasing their levels [26] in contradiction to our results where it displayed a nonsignificant positive correlation with both. This could be due to increased extraosseous production of sclerostin and Dkk-1 and hyperphosphatemia, increased calcitonin exposure, and absent renal clearance of both molecules [27, 28].

Both molecules did not correlate significantly to different treatments of HPT in our study (calcium supplement, active vitamin D and analogs, cinacalcet, sevelamer HCl, and parathyroidectomy) in contradiction to Kuczera et al., 2016, who reported an increase in sclerostin level (in 42 patients out of 58 included in their study) after cinacalcet therapy and related that to the decrease in PTH levels, but actually, the other 16 patients whose PTH levels did not decrease showed an increase in their sclerostin level over their study period [29] which means that further larger RCT are needed to clarify this dilemma.

In our study, sclerostin and Dkk-1 did not correlate significantly to Kt/v, calcium, phosphorus, ALP, iron, TSAT, TIBC, and ferritin. Both molecules correlated negatively to EPO treatment but only Dkk-1 showed significance (*r* = −0.372, *P*=0.003). This finding is overlooked and rarely found in the literature. Sharba and Al Zahid, 2016, found a positive correlation between EPO and sclerostin in their study on 21 male patients (12 on dialysis and 9 not on dialysis; duration of dialysis 20.9 ± 3.25 months) [30]. More detailed studies are needed to explore this interesting issue.

A study done by Stróżecki and colleagues concluded that patients with vascular calcification (VC) were older and had higher LVMI and no significant differences were found with respect to PTH, phosphorus calcium which is in accordance with our results. VC coexists with hypertrophy of the left ventricle especially when both valves are calcified. Even short-lived incidents involving increased product Ca x P can lead to cardiovascular calcification [31, 32].

LVH has a prevalence of approximately 40% in patients with chronic kidney disease (CKD), and it progressively increases with CKD progression up to 75% in ESRD patients [33, 34]. Foley et al. followed 596 patients on hemodialysis with no previous history of heart disease to determine whether the frequency of LVH correlates with the length of the dialysis. After 18 months of dialysis, the author recorded that 62% of patients had an increased LV mass index and 49% had developed LV failure [35]. CKD patients are faced with both pressure and volume overload states; however, sustained overload in combination with CKD-associated factors such as SHPT, RAAS activation, and anemia may result in maladaptive LVH until the final picture of uremic cardiomyopathy ensues [36–39].

Our results reveal that there was a significant negative correlation of sclerostin and Dkk-1 with left ventricular mass (LVM). LVM had a significant negative correlation with HDL, while it showed a significant positive correlation with age and a significant positive correlation with BSA, hypertension, AVC, MVC, and dry weight.

With linear regression analysis for LVM as a dependent factor, there was a nonsignificant prediction of age, serum sclerostin, aortic valve calcification, and mitral valve calcification for LVM; on the other hand, hypertension had a significant positive independent prediction for LVM, while serum HDL and serum DDK-1 were negative significant independent indicators for LVM.

As regards correlations with LVM/BSA (LVMI), there was a significant negative correlation of serum sclerostin and Dkk-1 with LVMI and a significant negative correlation with hemoglobin, albumin, midarm circumference, and dry weight with LVMI, while there was a highly significant positive correlation with age, AVC, and MVC with LVMI. With linear regression analysis for LVMI, there was a nonsignificant prediction of age, HDL, MVC, and serum sclerostin for LVMI; on the other hand, serum DDK-1 was a significant negative indicator for LVMI and hypertension was a weak significant positive predictor for LVMI (*P* value = 0.05).

Our results are in disagreement with Brovko and colleagues, 2018, who had done a study on 131 CKD Russian patients (stages 1–5, average age 42.4 ± 13.7 years) and revealed that, with univariate analysis, serum sclerostin levels correlated positively with LVM index (*r* = 0.545; *P* < 0.01) but with multivariate regression, it was negatively correlated to CVC and suggested that it may protect the heart and vessels against calcification with CKD advancement [40].

It is well known that Wnt-*β*-catenin signaling pathway inhibition by sclerostin and Dkk-1 protects against cardiac AV and MV calcifications and both have a significant negative correlation with both valves' calcification [41, 42]. Our study revealed that AVC and MVC had a significant negative correlation with sclerostin but a nonsignificant negative correlation with Dkk-1 which coincides with Yang et al., 2015, who reported that circulating sclerostin but not Dkk-1 is inversely associated with aortic calcifications and future cardiovascular events [43]. Krishna et al. reported that sclerostin decreases the expression of genes involved in matrix degradation and calcification and thereby inhibits atherosclerosis; it is likely that sclerostin could function as an inhibitor of vascular calcification [44].

On the contrary, other studies showed that elevated serum sclerostin levels were seen in patients with aortic valve calcification with increased upregulation of sclerostin mRNA [45]. Several elements could have contributed to these conflicting results. Studies investigated the presence of vascular calcification at different anatomical locations and used different statistical analyses (by adjusting for different potential confounding factors). In addition, differences in the time period between administration of enoxaparin (or other low molecular weight heparins that are used as anticoagulants) and blood collection could also be important, since enoxaparin stimulates the release of sclerostin into the circulation. This also implies that the heterogeneity of study populations—patients at different CKD stages, with or without dialysis treatment, whether or not they receive low molecular weight heparins—contributes to the observed inconsistency. Lastly, it is known that there are large discrepancies between sclerostin assays. The antibodies that are used in the distinct assays bind different epitopes; therefore, some antibodies will capture only the intact sclerostin molecule, while others might also bind to sclerostin fragments [46].

In our study, sclerostin and Dkk-1 had a significant negative correlation with LVM and LVM/BSA. Moreover, Dkk-1 appeared to be an independent indicator of LVM and LVM/BSA and they may be acting as protectors against LV hypertrophy or may be only associated with LV hypertrophy in CKD patients.

Van de Schans and colleagues demonstrated that interruption of Wnt signaling in the mice lacking the Dvl-1 gene delays the onset of pressure overload-induced cardiac hypertrophy. Therefore, the Wnt/Frizzled pathway may provide novel therapeutic targets for antihypertrophic therapy [47].

Because Wnt signaling is very complex and its effects may be quite varied according to the cell system, specific ligands/receptors involved, and timing of any interventions which presents a challenge to investigators, but it offers rich possibilities for new insights into cardiac pathophysiology and the identification of new therapeutic targets [48].

Limitations of this study include the relatively small population studied in a single-center and the cross-sectional design. However, to our knowledge, there are only limited studies addressing the relationship between Wnt pathway inhibitors and LVMI. More studies are needed to ascertain the obtained results.

## Figures and Tables

**Table 1:** Some demographic, clinical, and laboratory characteristics of the whole cohort.

Parameter	Study group (*n* = 65)	
Age (years)	46.14 ± 16.201	
Gender	Male	55.6%
	Female	44.4%
Dry weight (kg)	73.16 ± 15.89	
Midarm circumference (cm)	29.52 ± 4.25	
Waist circumference (cm)	98.21 ± 16.43	
BMI	26.53 ± 5.31	
BSA	1.82 ± 0.23	
Smoking	Current smoker	6 (10%)
	Ex-smoker	6 (10%)
	Nonsmoker	51 (80%)
HD duration (months)	35 (6.00–168)	
Hypertension	85.2%	
Diabetes	13.1%	
Coronary artery disease	9.8%	
Kt/v	1.13 ± 0.28	
Serum albumin (g/dl)	3.7262 ± 0 .43928	
Serum calcium (mg/dl)	8.3000 ± 0.92718	
Serum phosphorus (mg/dl)	4.9344 ± 1.58481	
iPTH (pg/ml)	49 (16–221)	
Alkaline phosphatase	145 (11–2152)	
HB (gm/dl)	9.3820 ± 1.65081	

kg: kilogram; cm: centimeter; BMI: body mass index; BSA: body surface are; Kt/v: kinetic time over volume; gm/dl: gram/deciliter; mg/dl: milligram/deciliter; iPTH: intact parathyroid hormone; pg/ml: picogram/millimeter; HG: hemoglobin.

**Table 2:** Comparative analysis of some demographic and echocardiographic data among the studied two subgroups.

Parameter	Subgroup 1	Subgroup 2	*P* value
Age (years)	37.61 ± 14.47	51.74 ± 14.93	0.001^*∗∗*^
BMI	27.018 ± 5.88	26.23 ± 4.99	0.573
BSA	1.835 ± .239	1.816 ± .220	0.754
HD duration (month)	31.00 (6.00–168)	35.50 (6.00–144)	0.318
DM	8.3%	16.2%	0.462
HTN	75.0%	87.2%	0.136
Midarm circ (cm)	30.50 ± 4.89	28.89 ± 3.71	0.150
Waist circ (cm)	97.71 ± 14.54	98.54 ± 17.74	0.849
Dry weight (kg)	74.04 ± 17.32	72.58 ± 15.11	0.729
Systolic BP (mmhg)	134.58 ± 16.41	144.05 ± 14.036	0.019^*∗*^
Diastolic BP (mmhg)	82.92 ± 9.55	87.84 ± 7.124	0.025^*∗*^
E/A	0.905 (.52–1.62)	0.760 (.35–1.65)	0.069
E_ declaration (m sec)	154.17 ± 37.13	164.04 ± 37.95	0.316
E1/A1	0.7900 (.36–1.88)	0.640 (.40–1.83)	0.237
IVRT (m sec)	76.75 ± 11.97	76.82 ± 9.98	0.980
IVCT (m sec)	71.13 ± 9.72	72.36 ± 9.04	0.611
ET (m sec)	285.17 ± 20.74	285.10 ± 17.37	0.990
MPI	0.517 ± .0463	0.523 ± .045	0.576
AVC	(0–2)	(0–4)	< 0.001^*∗∗*^
MVC	(0–2)	(0–4)	0.005^*∗*^

^*∗*^Significant at *P* ≤ 0.05. ^*∗∗*^Highly significant at *P* ≤ 0.001. BMI: body mass index; BSA: body surface area; HD: hemodialysis; DM: diabetes mellitus; HTN: hypertension; circ: circumference; cm: centimeter; kg: kilogram; E/A: early diastolic mitral inflow velocity/late diastolic mitral inflow velocity; E1/A1: early diastolic mitral annular velocity/late diastolic mitral annular velocity; IVCT: isovolumetric contraction time; m sec: millisecond; IVRT: isovolumetric relaxation time; MPI: myocardial performance index; AVC: aortic valve calcification; MVC: mitral valve calcification.

**Table 3:** Comparative analysis of some laboratory data among the studied two subgroups.

Parameter	Subgroup 1	Subgroup 2	*P* value
HB (gm/dl)	10.096 ± 1.43	8.92 ± 1.633	0.006^*∗*^
Iron (µg/dl)	82.5 (36.00–202)	72 (35.00–222)	0.087
TSAT%	38 (13.00–85.00)	32 (17.00–91.00)	0.111
TIBC	225.83 ± 36.58	221.08 ± 45.195	0.668
S. albumin (gm/dl)	3.85 ± .295	3.64 ± .498	0.066
Alk_phosph (IU/L)	131 (11.00–2152)	168 (64.00–1003)	0.209
S. chol. (mg/dl)	162 (93.0–243)	130 (79.0–375)	0.140
S. TG (mg/dl)	135.5 (48.0–232)	112 (45.0–276)	0.438
S. HDL (mg/dl)	25 (16.0–63.0)	23 (11.0–56.0)	0.169
S. LDL (mg/dl)	104 (29.8–159.8)	87.4 (39.8–332.2)	0.232
Kt/V	1.12 ± .34	1.13 ± .23	0.877
S. Ca (mg/dl)	8.47 ± .976	8.19 ± .89	0.250
S. PO_4_ (mg/dl)	4.92 ± 1.45	4.95 ± 1.69	0.945
iPTH (pg/ml)	648 (10.60–1900)	576 (27.00–2000)	0.790
Sclerostin (ng/ml)	35.25 (3.40–59.3)	7.70 (2.10–60.4)	0.013^*∗*^
Dkk-1 (pg/ml)	63 (12.00–132)	33 (13.00–112)	0.011^*∗*^

^*∗*^Significant at *P* ≤ 0.05. ^*∗∗*^Highly significant at *P* ≤ 0.001. HB: hemoglobin; gm/dl: gram/deciliter; *µ*g/dl: microgram/deciliter; TSAT: transferrin saturation, Alk_phosph: alkaline phosphatase; IU/L: international unit/liter; S. chol.: serum cholesterol; S. TG: serum triglycerides; S. HDL: serum high-density lipoprotein; S. LDL: serum low-density lipoprotein; Kt/V: kinetic time/volume; S. Ca: serum calcium; S. PO_4_: serum phosphorus; iPTH: intact parathyroid hormone; pg/ml: picogram/milliliter; ng/ml: nanogram/milliliter.

**Table 4:** Correlation between LVM and LVM/BSA and some demographic and laboratory parameters among the studied subgroups.

	LVM		LVMI	
	*r*	*P*	*r*	*P*
S. sclerostin (ng/ml)	−0.329	0.010^*∗*^	−0.257	0.046^*∗*^
S. Dkk-1 (pg/dl)	−0.427	0.001^*∗∗*^	−0.324	0.012^*∗*^
Age (years)	0.409	0.001^*∗∗*^	0.352	0.007^*∗*^
BMI	0.219	0.085	−0.199	0.118
BSA	0.278	0.027^*∗*^	−0.305	0.015^*∗*^
HD_duration	−0.153	0.264	0.015	0.979
Gender	−0.112	0.380	0.153	0.232
HTN	0.300	0.019^*∗*^	0.247	0.054
HB (gm/dl)	−0.245	0.057	−0.299	0.019^*∗*^
S. albumin (gm/dl)	−0.229	0.076	−0.294	0.021^*∗*^
Syst_BP (mmhg)	0.243	0.059	0.249	0.053
Diast BP (mmhg)	0.251	0.051	0.211	0.103
S. cholesterol	−0.126	0.334	−0.205	0.112
S. TG (mg/dl)	−0.024	0.855	−0.179	0.166
S. HDL (mg/dl)	−0.268	0.036^*∗*^	−0.161	0.214
S. LDL (mg/dl)	−0.076	0.563	−0.158	0.225
AVC	0.417	0.001^*∗∗*^	0.464	< 0.001^*∗∗*^
MVC	0.297	0.02^*∗*^	0.360	0.004^*∗*^
Mid_arm_circ (cm)	0.067	0.609	−0.423	0.001^*∗∗*^
Waist_circ (cm)	0.204	0.115	−0.128	0.326
Dry_weight (kg)	0.282	0.028^*∗*^	−0.286	0.026^*∗*^
Kt/V	−0.046	0.723	0.183	0.158
S. Ca (mg/dl)	−0.085	0.514	−0.074	0.573
S. PO_4_ (mg/dl)	0.242	0.060	−0.028	0.831
iPTH (pg/dl)	0.023	0.787	−0.035	0.861

Spearman's correlation used. ^*∗*^Significant at *P* ≤ 0.05; ^*∗∗*^highly significant at *P* ≤ 0.001. LVM: left ventricular mass; LVM/BSA: left ventricular mass/body surface area; S. Dkk-1: serum Dickkopf-1; BMI: body mass index; BSA: body surface area; HD: hemodialysis; HTN: hypertension; HB (gm/dl): hemoglobin concentration (gram/deciliter); Syst_BP: systolic blood pressure; Diast BP: diastolic blood pressure; mmhg: millimeter Mercury; S. TG; serum triglyceride; S. HDL: serum high-density lipoprotein; S. LDL: serum low-density lipoprotein; AVC: aortic valve calcification; MVC: mitral valve calcification; circ: circumference; Kt/V: kinetic time/volume; S. Ca: serum calcium; S. PO_4_: serum phosphorus; iPTH: intact parathyroid hormone; mg/dl: milligram/deciliter; pg/dl: picogram/deciliter; ng/dl: nanogram/deciliter.

**Table 5:** Linear regression analysis of LMV with some parameters among the studied subgroups.

Parameter	Unstandardized coefficients		Standardized coefficients	*P* value^*∗*^	95.0% confidence interval for B	
	B	Std. error	Beta		Lower bound	Upper bound
S (constant)	214.80	27.39		< 0.001	159.72	269.88
Age	0.73	0.408	0.216	0.079	−0.088	1.55
HTN	40.65	14.89	0.279	0.009	10.70	70.50
HDL	−1.22	0.550	−0.232	0.031	−2.33	−0.117
AVC	9.62	6.287	0.227	0.132	−3.015	22.268
MVC	1.94	6.00	0.043	0.748	−10.13	14.014
S. sclerostin	0.049	0.325	0.018	0.882	−0.605	0.703
Dkk-1	−0.58	0.189	−0.354	0.003	−0.963	−0.204

Dependent variable: LVM. ^*∗*^Significant *P* ≤ 0.05. LVM: left ventricular mass; HTN: hypertension; HDL: high-density lipoprotein; AVC: aortic valve calcification; MVC: mitral valve calcification; Dkk-1: Dickkopf-1.

**Table 6:** Linear regression analysis of LMV/BSA with some parameters among the studied subgroups.

Parameter	Unstandardized coefficients		Standardized coefficients	*P* value^*∗*^	95.0% confidence interval for B	
	**B**	Std. error	Beta		Lower bound	Upper bound
S (constant)	114.11	16.88		< 0.001	80.16	148.06
Age	0.189	0.251	0.101	0.455	−0.316	0.694
HTN	18.43	9.18	0.229	0.050	−0.025	36.80
HDL	−0.436	0.339	−0.150	0.205	−1.118	0.246
AVC	7.638	3.876	0.326	0.055	−0.155	15.430
MVC	2.320	3.701	0.092	0.534	−5.121	9.761
S. sclerostin	0.079	0.201	0.053	0.695	−0.324	0.482
DKK	−0.244	0.116	−0.268	0.041^*∗*^	−0.478	−0.010

Dependent variable: LVM/BSA. ^*∗*^Significant at *P* ≤ 0.05. LVM/BSA: left ventricular mass/body surface area; HTN: hypertension; HDL: high-density lipoprotein; AVC: aortic valve calcification; MVC: mitral valve calcification; Dkk-1: dickkopf-1.

**Table 7:** Correlation between AVC, MVC, iPTH, and some clinical and chemical variables.

	AVC		MVC		iPTH	
	*r*	*P*	*r*	*P*	*r*	*P*
Age	0.55	<0.0001^*∗*^	0.38	0.003^*∗*^	−0.05	0.72
HD duration	0.23	0.1	0.01	0.95	0.24	0.08
Ferritin	−0.09	0.48	−0.05	0.69	−0.006	0.96
Hemoglobin	−0.22	0.09	−0.18	0.16	0.22	0.09
Iron	−0.1	0.47	−0.08	0.5	0.12	0.35
TIBC	0.04	0.78	−0.13	0.33	−0.01	0.93
TSAT	−0.09	0.5	−0.05	0.72	0.08	0.55
Albumin	−0.15	0.24	−0.17	0.2	0.18	0.16
Syst. BP	0.12	0.35	0.04	0.75	0.06	0.66
Diast. BP	0.1	0.47	0.08	0.54	−0.01	0.93
Cholesterol	−0.17	0.19	−0.27	0.04^*∗*^	0.03	0.82
HDL	0.06	0.65	0.09	0.5	0.04	0.78
LDL	−0.17	0.19	−0.28	0.03^*∗*^	0.02	0.86
Kt/v	−0.01	0.91	−0.12	0.36	0.03	0.82
Calcium	−0.02	0.89	0.09	0.52	0.03	0.8
Phosphorus	−0.09	0.48	−0.04	0.75	0.41	0.001^*∗*^
Sclerostin	−0.47	<0.0001^*∗*^	−0.27	0.04^*∗*^	0.15	0.24
Dkk-1	−0.15	0.25	−0.16	0.21	0.03	0.8
iPTH	−0.18	0.18	−0.03	0.85		

Spearman's correlation used. ^*∗*^Significant when *P* < 0.05.

**Table 8:** Correlation between Dkk-1, sclerostin, and some clinical chemical variables.

		Dkk-1	Sclerostin
HPT_ttt	*r*	0.041	0.024
	*P*	0.754	0.854

Kt_V	*r*	0.155	0.158
	*P*	.233	0.224

S. Ca	*r*	0.023	0.106
	*P*	0.857	0.415

S. PO_4_	*r*	−0.089	−0.005
	*P*	0.494	0.971

Alk_phosphatase	*r*	0.001	0.108
	*P*	0.994	0.408

TSAT	*r*	0.059	0.197
	*P*	0.654	0.128

TIBC	*r*	−0.029	−0.105
	*P*	0.825	0.420

Iron	*r*	0.020	0.091
	*P*	0.880	0.484

S. ferritin	*r*	0.109	0.174
	*P*	0.401	0.180

Erythropoietin	*r*	−0.372	−0.134
	*P*	0.003^*∗*^	0.303

Spearman's correlation used. ^*∗*^Significant when *P* < 0.05. HPT_ttt = hyperparathyroidism treatment. TIBC = total iron-binding capacity.

## Data Availability

All data analyzed during this study are included within this manuscript.
